# Evolution under Fluctuating Environments Explains Observed Robustness in Metabolic Networks

**DOI:** 10.1371/journal.pcbi.1000907

**Published:** 2010-08-26

**Authors:** Orkun S. Soyer, Thomas Pfeiffer

**Affiliations:** 1 Systems Biology Program, School of Engineering, Computing and Mathematics, University of Exeter, Exeter, United Kingdom; 2 Program for Evolutionary Dynamics, Harvard University, Cambridge, Massachusetts, United States of America; University of Washington, United States of America

## Abstract

A high level of robustness against gene deletion is observed in many organisms. However, it is still not clear which biochemical features underline this robustness and how these are acquired during evolution. One hypothesis, specific to metabolic networks, is that robustness emerges as a byproduct of selection for biomass production in different environments. To test this hypothesis we performed evolutionary simulations of metabolic networks under stable and fluctuating environments. We find that networks evolved under the latter scenario can better tolerate single gene deletion in specific environments. Such robustness is underlined by an increased number of independent fluxes and multifunctional enzymes in the evolved networks. Observed robustness in networks evolved under fluctuating environments was “apparent,” in the sense that it decreased significantly as we tested effects of gene deletions under all environments experienced during evolution. Furthermore, when we continued evolution of these networks under a stable environment, we found that any robustness they had acquired was completely lost. These findings provide evidence that evolution under fluctuating environments can account for the observed robustness in metabolic networks. Further, they suggest that organisms living under stable environments should display lower robustness in their metabolic networks, and that robustness should decrease upon switching to more stable environments.

## Author Summary

One of the most surprising recent biological findings is the high level of tolerance organisms show towards loss of single genes. This observation suggests that there are certain features of biological systems that give them a high tolerance (i.e. robustness) towards gene loss. We still lack an exact understanding of what these features might be and how they could have been acquired during evolution. Here, we offer a possible answer for these questions in the context of metabolic networks. Using mathematical models capturing the structure and dynamics of metabolic networks, we simulate their evolution under stable and fluctuating environments (i.e., available metabolites). We find that the latter scenario leads to evolution of metabolic networks that display high robustness against gene loss. This robustness of *in silico* evolved networks is underlined by an increased number of multifunctional enzymes and independent paths leading from initial metabolites to biomass. These findings provide evidence that fluctuating environments can be a major evolutionary force leading to the emergence of robustness as a side effect. A direct prediction resulting from this study is that organisms living in stable and fluctuating environments should display differing levels of robustness against gene loss.

## Introduction

High-throughput single gene deletion studies in several organisms revealed that a large fraction of genes have little or no detectable fitness effects when compromised [1]–[5]. These observations raise the question of how biological systems can acquire and maintain such robustness against gene loss. As for any biological trait, robustness could be adaptive, resulting from direct selection for it, or non-adaptive, resulting as a byproduct of other selective pressures [6]. Understanding which of these modes apply is important both to distill the design principles of biological systems and to understand how amenable robustness is to manipulation [7].

Direct selection for robustness against gene loss is expected to be weak [8], becoming relevant only under high mutation rates [9], [10]. In line with these theoretical findings, empirical analyses find only limited contribution of gene duplications to the observed robustness [11]–[15]. On the other hand, different forms of robustness are shown to evolve in non-adaptive fashion under certain conditions. For example, in near-neutral fitness landscapes mutational robustness can emerge easily [16]. In metabolic networks, it is argued that properties of enzyme kinetics can render the systems robust against partial loss-of-function mutations [17], [18]. Moreover, robustness against small mutations is shown to evolve in gene regulatory networks selected for dynamic stability [19], [20] and robustness against gene deletions is shown to evolve in signaling networks under parasite interference [21].

It is possible that biomass production and adaptation to multiple environments act as similarly realistic selective pressures on metabolic networks that could lead to the emergence of robustness as a byproduct. The former can drive the emergence of isoenzymes for increased dosage [22], resulting in a clear case of functional redundancy mediated robustness. The latter could lead to multiple pathways, each specializing in processing metabolites present in one of the multiple environments. These multiple pathways could compensate for each other, particularly, in rich media [7]. This scenario is in line with the observation that the estimated fraction of dispensable genes at both metabolic [23]–[25] and genome scale [26] reduces dramatically when multiple environments are considered. The most recent computational analysis of metabolic networks from *Escherichia coli* and *Saccharomyces cerevisiae* finds that, when the effect of deletion is tested in all possible environments, only half of all reactions determined to be dispensable under rich media could be considered dispensable for “real” [25]. Further, almost all of the remaining cases can be explained by recent duplications, horizontal gene transfer events or pleitropic effects (i.e. compensation by multifunctional enzymes) [25]. It is important to note that these studies typically judge dispensability based on stoichiometric approaches such as flux balance analysis (FBA). By focusing only on lethal knockouts, and ignoring the fitness effect of non-lethal ones, these approaches therefore overestimate robustness.

**Figure 1:**
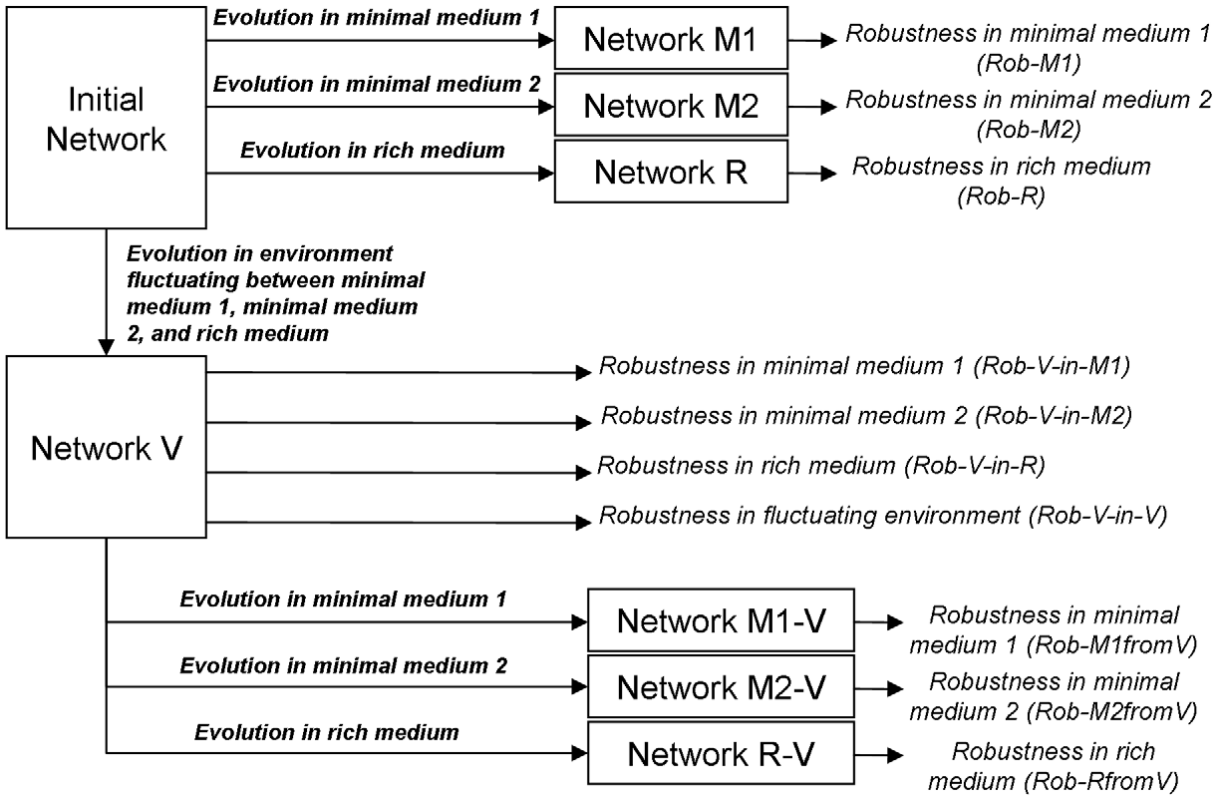
Analysis scheme. To investigate the evolution of robustness against knock-outs we simulate evolution of metabolic networks in different environmental scenarios and under selection for rate of biomass formation. We consider three constant environments containing either minimal medium 1, minimal medium 2 or rich medium, and a fluctuating one that switches between these three media. The resulting networks are referred to as network M1, M2, R, and V, respectively. The networks are tested for robustness by determining the fitness of knockouts. The three networks from the constant environments (M1, M2, R) are tested in the environment where they evolved. The network from the fluctuating environment (network V) is tested individually in each of the three media it adapted to during evolution, and over all three media. In summary, we have four different sets of evolved networks (M1, M2, R, V) and seven different distributions of fitness values of knockouts (Rob-M1, Rob-M2, Rob-R, Rob-V-M1, Rob-V-M2, Rob-V-R, Rob-V-V). To test whether differences in robustness between networks from constant and from fluctuating environments are transient, the network from the fluctuating environment is subsequently evolved in the three constant environments, and the emerging networks are tested for robustness. This gives three additional sets of evolved networks (VM1, VM2, VR), and three additional distributions characterizing their robustness (Rob-M1fromV, Rob-M2fromV, Rob-RfromV).

Taken together, the above described studies suggest that observed robustness against gene deletion in metabolic networks is a byproduct of their evolutionary dynamics under changing environmental conditions. Early studies on the effects of changing environments in evolution have shown that it can facilitate polygenic variation [27], [28] and can lead to modularity at network level [29]–[32]. In addition, abrupt changes in selective pressure are shown to lead to significant changes in metabolic networks [33]. Here, we specifically study the effects of fluctuating selection on the emergence of robustness in metabolic networks. Using a well-accepted scenario of duplication and specialization [34]–[37], we simulated evolution of metabolic networks under selection for converting environmentally available metabolites into biomass. These simulations started from initial networks composed of unspecific enzymes, which duplicate and specialize as evolution progresses, resulting in metabolic networks with high biomass production rate. To test the effect of the environment on the properties of evolved networks we performed simulations under stable and fluctuating environments (Figure 1). Networks that evolved in a stable environment were selected for biomass production in either one of two different minimal media or in a rich medium; network fitness was a function of biomass production rate given the metabolites in the media. Networks that evolved in a fluctuating environment faced changes between these three media and were selected for biomass production in all of them; network fitness was defined as the geometric mean of fitness values in each of the individual media. The resulting networks were tested for their robustness against gene loss. For networks that evolved in a fluctuating environment, robustness was determined separately in each medium and over all media allowing us to investigate whether any resulting robustness in these networks is apparent or real. A detailed schematic of the simulations and analysis is given in Figure 1.

## Results

To study the effect of selection under fluctuating environments on metabolic network properties, we relied on a proposed evolutionary scenario [35]. According to this scenario, metabolic networks characterized by large numbers of enzymes with high specificity have evolved from ancestral networks consisting of few enzymes with broad specificity [34], [37]. Such evolution could be driven by selection for increased growth rate (i.e. biomass production rate), and mutations affecting kinetic properties of enzymes and resulting in gene duplications. Although a number of alternative scenarios for the evolution of novel enzymes and metabolic pathways have been proposed [38], this scenario is plausible for the early evolution of metabolic networks.

Here, we implement this scenario using a computational model of metabolic networks. In brief, the model consists of metabolites, enzymes that catalyze the transfer of biochemical groups between metabolites, and transporters that can allow intake and release of metabolites (see *Methods*). We start evolutionary simulations with enzymes that can catalyze all group transfer reactions. In the course of evolution enzymes can subsequently specialize through duplications and mutations. This process is driven by the assumption that there is a trade-off between catalytic activity and specificity. This assumption is well supported by the existence of specialized enzymes in nature and by several directed evolution experiments that exploit such trade-off for protein engineering [37]–[39]. The model structure allows us to capture both subfunctionalization [40], [41] and neofunctionalization [42]; two processes that are believed to be at the core of evolution of gene duplicates [43]–[46]. Running evolutionary simulations that mimic natural evolution as a deterministic process we evolve networks towards a local optimum and analyze the aspects in which these optima differ for different fitness landscape. The deterministic approach to simulating evolution corresponds to a scenario with a large population and low mutation rate (also referred to as strong selection - weak mutation scenario [47]). In summary, the presented model captures the dynamics and stoichiometry of metabolic networks and the evolution of these properties. Previously, we have shown that it can result in the evolution of complex metabolic networks that have very similar global properties to their natural counterparts [36].

**Figure 2:**
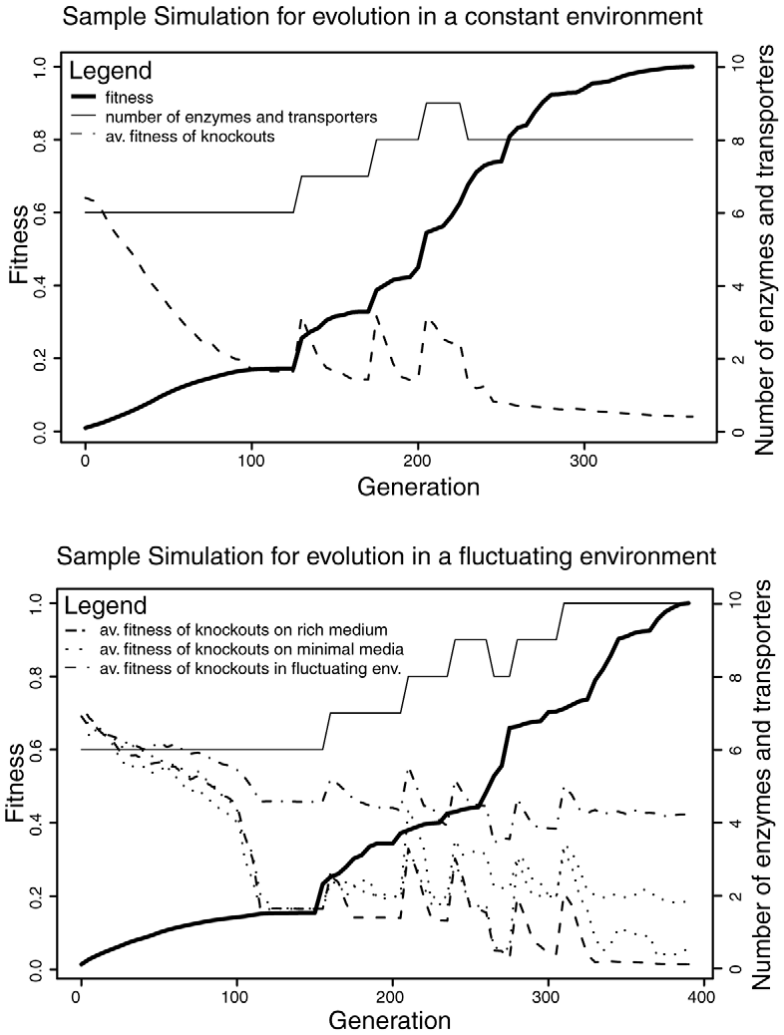
Results from sample M1 and V simulations. The plot shows the number of unique transporters and enzymes, network fitness (relative to final fitness), and the average fitness of a knockout (i.e. robustness) over generations. Initially robustness is high because the ancestral network contains enzymes with broad specificity, which can compensate for each other. As enzymes specialize fitness increases and robustness decreases in general. Whenever an enzyme or transporter duplicates (as at generation 120, 170 and 190 for the M1 run), the robustness increases because the two copies initially cover the same reactions. As the copies diverge in function, their contribution to robustness becomes smaller and smaller. The simulation of evolution in the fluctuating environment (lower panel) shows that although robustness over all environments decreases over time, robustness is maintained to a considerable degree on each of the three media, in particular the rich one. The resulting networks from these simulations are shown in Figure 3.

**Figure 3:**
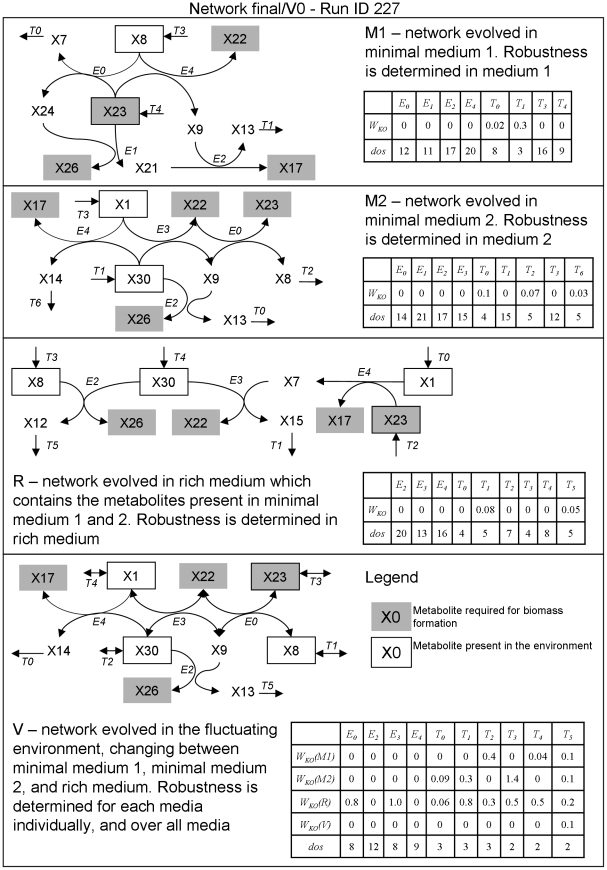
Structure, reaction kinetics and knockout effects for sample networks resulting from evolution under three different stable environments (M1, M2, and R) and one that fluctuates over these three (V). Metabolites constituting biomass are shown with a gray backdrop, while metabolites taken from the medium are shown in a black box. For example, network M1 takes up metabolites X8 and X23 (in binary notation, metabolites 01000 and 10111) from the media and uses a network of 4 enzymes and 4 transporters in order to produce biomass metabolites X17, X22, X23 and X26 (in binary notation, metabolites 10001, 10110, 10111, and 11010). The net reaction of the network is 2×01000+4×10111→biomass +00111+01101. The latter two metabolites are the waste products X7 and X13. Note that in this sample run, one of the metabolites required for biomass formation happens to be present in the environment. The table shows that most knockouts are lethal in this network. Only transporters T0 and T1, which excrete the waste products X7 and X13 respectively, can be knocked out. Even then, the knockout infers large fitness costs as without the transporters the waste metabolites accumulate in the cell and strongly inhibit growth. Network M2 uses X1 and X30 for biomass formation. The resulting network consists of 4 enzymes and 5 transporters. X8, X13 and X14 are excreted as waste products. The rich medium combines the resources available in the two minimal media.

**Figure 4:**
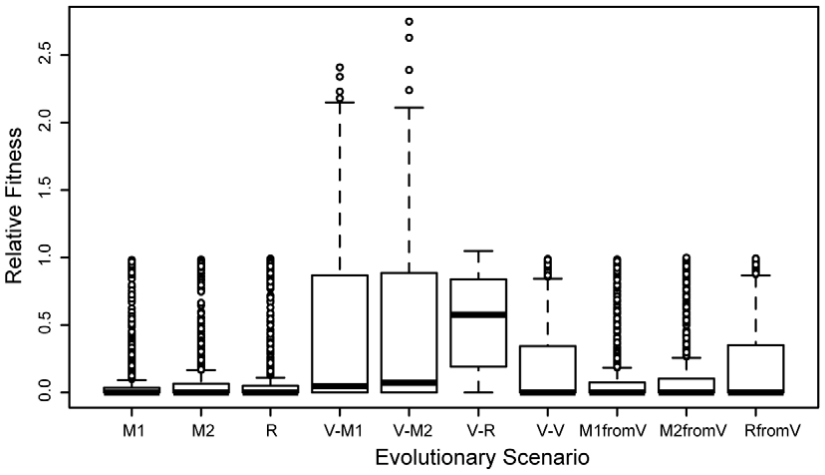
Distribution of relative fitness for single knockouts in networks resulting from different evolutionary scenarios. Each distribution contains measurements from 100 networks and is shown as a boxplot, as implemented in the statistical package “R” (www.r-project.org). See legend of Figure 1 for analysis and naming details. To statistically analyze differences between the distributions, we performed pair-wise Kolmogorov-Smirnov tests. As expected, differences between equivalent distributions were statistically not significant (M1 vs. M2: p≈0.6; M1fromV vs. M2fromV: p≈0.3; V-M1 vs. V-M2: p≈0.7). The fitness distribution for R networks is highly similar to M1 and M2 (p≈0.96 and 0.8, respectively). The distributions M1fromV and M2fromV are similar to the distributions M1, M2 and R, with indication for statistically significant differences: Four of the pair-wise comparisons yield p-values larger than 0.1; while two comparisons yield p-values below 0.05 (M2fromV vs. M1: p≈0.026; M2fromV vs. R p≈0.008). All other pair-wise comparisons show statistically highly significant differences, with all p-values smaller than 0.0001.

Using this model we have run evolutionary simulations under different environmental scenarios (see *Methods*). In particular, we have evolved metabolic networks under three stable environments and a fluctuating one (Figure 1). In all these environments fitness was related to the ability of the network to convert available metabolites into biomass (see *Methods*). The three stable environments respectively contained either one of two randomly chosen pairs of metabolites (minimal media; M1 and M2) or both of them (rich media; R = M1 + M2). The fluctuating environment was assumed to vary between these three media. In all these simulations network fitness increased quickly as evolution progressed, and enzymes became more specialized (Figures 2 and 3). To understand how evolution under these different scenarios affected network robustness, we have analyzed the effect of single gene knockouts on fitness. As shown in Figure 4, we found that in networks evolved under fluctuating media, single gene deletions had significantly lower fitness effects compared to networks that evolved in stable media. Interestingly, the difference in robustness against gene deletion was most prominent when fitness was measured under rich media and was completely lost when it was measured over all media seen during evolution (see also Figure 2). Hence, fluctuating evolution resulted in the emergence of an “apparent” robustness against gene deletion that became most detectable in rich media.

To test that these results are robust against the main assumptions of the model and the simulation scheme, we have analyzed an alternative model. In this model, enzymes were allowed to maintain broader activity by introducing a small background rate for all reactions an enzyme can catalyze (see *Methods*). This approach is inline with the idea of “underground metabolites” [38] and allows us to start or continue an evolutionary simulation from any starting network. We use this ability to change the simulation scheme so that we start simulations under fluctuating environments from networks that already have evolved under stable environments. This alternative approach is potentially more inline with conditions in nature where networks can experience sudden changes in environmental conditions [33]. We find qualitatively the same results as with the previous analysis; robustness of networks evolved under a stable environment increase when they further evolve under fluctuating environments (Figure S2). As before, this higher robustness is apparent, however it is not completely lost when considering all environments a network experiences during evolution (Figure S2).

To understand the basis of such robustness we have analyzed the structure of networks resulting from evolution under stable and fluctuating media. As mentioned above, all evolutionary simulations resulted in enzymes that are specialized and in networks with faster biomass production compared to the ancestral ones. However, networks evolved under fluctuating media displayed two important features that distinguished them from networks evolved under stable media. Firstly, fluctuating environments resulted in networks that contain more redundant paths. The average number of independent fluxes (see *Methods*) that can be channeled through the network ranged from 1.2 to 1.4 for networks that evolved in stable environments, while it was 4.1 for networks evolving in fluctuating environment (Table 1). The extent of redundant paths in the latter networks is clearly seen in sample networks shown in Figure 3. Secondly and related, networks evolved under fluctuating environment contained significantly more multifunctional enzymes, i.e. enzymes that catalyzed more than one group transfer reaction (see *Methods*). Of the 100 independent simulations for each scenario, 72% of networks that evolved under fluctuating environments contained at least one multifunctional enzyme compared to 36%, 40% and 28% of networks evolved under stable environments M1, M2, and R respectively. Further, in networks evolved under fluctuating environment 24% of all enzymes were multifunctional, while only 6–9% were multifunctional in networks evolved under stable environment (Table 1).

**Table 1:** Network properties and robustness compiled from all networks resulting from 100 simulations for each of the evolutionary scenarios.

	M1	M2	R	V	M1fromV	M2fromV	RfromV
N Independent Fluxes	1.3	1.4	1.2	4.1	1.4	1.5	1.6
N Enzymes	545	542	509	724	596	600	587
N Multifunctional Enzymes	48	49	35	177	21	32	31
Avg. Robustness per Multifunctional Enzyme	0.890	0.874	0.935	0.677	0.886	0.929	0.893
Avg. Robustness per Monofunctional Enzyme	0.078	0.104	0.127	0.416	0.210	0.111	0.134
Avg. Robustness For Networks With Multifunctional Enzyme	0.281	0.297	0.338	0.614	0.263	0.313	0.427
Avg. Robustness For Networks Without Multifunctional Enzyme	0.046	0.070	0.096	0.382	0.082	0.102	0.242

The columns labeled M1, M2, R, and V display results from networks evolved under the two minimal media, the rich media and the fluctuating media respectively. The last three columns show results of continued evolution of those networks, which were obtained under fluctuating evolution, in stable media M1, M2 and R. Multifunctional enzymes are defined as those, which can catalyze more than one group transfer reaction (see *Methods*). Shown robustness values for networks evolved under fluctuation environment are those measured under rich media.

These clear differences in the global properties of networks evolved under fluctuating and stable media suggest that both the number of independent fluxes and the number of multifunctional enzymes in a network contribute to its robustness. To better understand the relation between these properties and robustness, we performed a detailed analysis of the fitness effects of single gene deletions (Table 1). In networks evolved under stable environments, the deletion of monofunctional enzymes had, on average, 7 to 11 fold larger effect compared with the deletion of multifunctional enzymes. Consequently, networks that contained multifunctional enzymes were on average 3 to 6 fold more robust compared to networks without any such enzymes.

Deletion of multifunctional enzymes might result in lower fitness effects either because these enzymes behave as isoenzymes (i.e. back up the function of another enzyme) or catalyze reactions that are non-essential but beneficial. We find evidence for both of these possibilities. Firstly, almost all multifunctional enzymes have low dosage, suggesting that the reactions they catalyze are non-essential but beneficial when they occur at low rate. Because these reactions are beneficial when occurring at low rate, there is no selective pressure for the multifunctional enzymes to duplicate (so to increase dosage) and potentially specialize. Secondly, most multifunctional enzymes have functions that overlap with other enzymes in the network. This finding results from a structural analysis of all networks evolved under one of the stable environments (M1-networks) and under fluctuating environments (V-networks) and that contain a multifunctional enzyme: We find that among the 36 M1-networks with multifunctional enzymes, 19 contain at least one multifunctional enzyme that behaves as an isoenzyme. Among the 72 V-networks with multifunctional enzymes, 42 contain at least one multifunctional enzyme that behaves as an isoenzyme. In both M1- and V-networks, multifunctional enzymes in the remaining networks catalyze at least one reaction that is not directly involved in biomass production, further supporting their non-essential role.

Interestingly, the difference in fitness effects of deleting multi- vs. mono-functional enzymes were significantly reduced in networks evolved under fluctuating environments. Considering fitness effects in different media, deleting monofunctional enzymes in these networks had, on average, only 1–2 fold larger effect compared with deleting multifunctional enzymes. Similarly, networks containing multifunctional enzymes were only 1 to 3 fold more robust than those without any such enzymes. These analyses suggest that while multifunctional enzymes can contribute significantly to robustness against gene deletion, evolution under fluctuating media increased robustness of networks mostly through generation of independent fluxes.

If evolution under fluctuating environments is the driving force behind emergence of robustness against gene deletion, would it be possible that robustness is lost as the environment stabilizes? Indeed, redundant paths (i.e. independent fluxes) might infer a cost on the organism due to increased number of enzymes that are not always required, or because some of the paths are in fact disadvantageous in some environments. This is the case in our simulations as we find certain gene deletions to increase fitness above wild type levels in networks evolved in fluctuating media (Figure 4). To further analyze the possibility of loosing robustness in stable environments we take networks evolved under fluctuating media and continue their evolution under any of the three stable media. As shown on Figure 4, we find that such subsequent evolution results in complete loss of any gained robustness against gene deletion; the distribution of fitness effects of gene deletions for these networks is the same as for those which have evolved in stable media originally. This reduction in robustness is accompanied by a reduction in both the number of multifunctional enzymes and independent fluxes (Table 1).

The finding that a switch in the environment towards stability leads to reduction in robustness fits nicely with the observation that prokaryotes specializing on one mode of energy generation has much reduced fraction of dispensable genes compared to generalists [48], however, it should be taken with care as we model switch to a stable environment to be perfect while in reality it is possible that environments are never entirely stable. It can be shown that even very rare fluctuations could maintain functional redundancy mediated robustness; for example, a gene providing a fitness advantage of *s* in a given environment could be maintained even if that environment is seen only once every *s*/*u* generations, where *u* is the mutation rate [25].

## Discussion

Here we have provided evidence that fluctuating environments can lead to emergence of robustness against gene loss in metabolic networks. Using computer simulations that embed a plausible scenario of metabolic network evolution, we found that selection for biomass production rate in a fluctuating media leads to emergence of networks, which can tolerate single gene deletions more readily. This robustness against gene loss is highest when fitness is measured under rich media, where all metabolites seen during evolution are considered to be available, and diminishes as fitness is measured separately under each media. We find that the molecular basis of such robustness in evolved networks is an increase in the number of independent fluxes and multifunctional enzymes.

These findings are perfectly in line with observations made in natural, current-day metabolic networks. Computational analysis of metabolic networks from *E. coli* and *S. cerevisiae* finds that most of the observed robustness in rich media is apparent, strongly diminishing as different environments are considered separately [23]–[25]. While these works have suggested that such robustness could be due to compensating pathways and to enzymes that have differential efficiencies under different environmental conditions [23], [25], the presented study provides a clear evolutionary route to these features. Further, it indicates that considering dynamic response of metabolic networks might reveal more severe fitness effects of gene deletions when considering multiple environments.

Interestingly, we find that robustness and its underlying features would be lost entirely as the environment stabilizes and network evolution continues. This leads to the prediction that robustness of the metabolic network in an organism should be directly correlated with environmental conditions it experiences; organisms whose metabolism depends on stable resources should display lower robustness. This prediction is supported both from a specific gene deletion study in *Mycoplasma genitalium* [3], which has a minimal metabolism, and from a larger comparison of gene dispensability in specialist and generalist prokaryotes [48].

Any attempt to fully distill design principles of biological systems has to consider evolutionary dynamics [49]. This can be achieved with *in silico* evolution as presented here or alternatively by considering the space of possible metabolic networks and how evolution could move in this space. The two approaches are complementary; recent modeling studies using the latter approach are providing us with important insight on common design principles that can result in evolution [50], [51], while approaches like the one presented here show how different selective pressures can shape the global properties of metabolic networks. As with any modeling study, the presented analysis has limitations and potential caveats. In particular, our analysis was limited in network size due to computational costs associated with evolutionary simulations and the generic model and the measure of robustness had to be based on several simplifications and assumptions about metabolism. While we find that our main findings are robust against such limitations and the main modeling choices, a full confirmation of our results can only be achieved with experimental studies. In this regard, we note that long-term evolution experiments under stable lab conditions provide a direct test bed to confirm the ideas presented here. These studies have already shown that evolution under stable environments reduce the metabolic breadth of *E. coli* [52]. We would expect that it has also reduced its “apparent” robustness against gene loss.

## Methods

Methods have been described in detail previously [36]. In brief, we implement a well-accepted scenario of metabolic network evolution [35], where an ancestral network composed of few unspecific enzymes evolves through mutations altering kinetic rates and duplications. At the core of this scenario is the argument that new enzyme activities result from specialization of enzymes with broad activity [34]. There is now empirical evidence that such specialization have led to the evolution of most, if not all, enzyme superfamilies [37]. In addition, laboratory evolution has been successfully employed to select or de-select for promiscuous functions, thereby altering enzyme function(e.g. [38], [39]).

The details of different modeling choices we made are as follows.

### Metabolites

Metabolites are assumed to consist of five different biochemical groups. Each biochemical group is either present once or is absent, resulting in a total of 32 possible metabolites. Each metabolite can be represented by a binary string of length 5, where “1” at position *g* denotes the presence of group *g*, whereas “0” denotes the absence of that group. Metabolites are associated with a random free energy that is taken from a uniform distribution between zero and one, and that is required to specify thermodynamic properties of the biochemical reactions. For the production of biomass, it is necessary to have at least one donor and acceptor of each group as external metabolites. Thus, a minimal medium contains two randomly chosen metabolites as a donor-acceptor pair. Rich medium consists of two different random donor-acceptor pairs. Four randomly chosen metabolites are involved in biomass formation. All of these random choices are made independently for each evolutionary simulation.

### Enzymes

Enzymes catalyze the transfer of a specific biochemical group. We assume that groups are transferred by a “ping-pong mechanism”: A donor of a group transfers the group to the appropriate enzyme and is thereby transformed into its corresponding acceptor. The enzyme then transfers the group to an acceptor, thereby transforming it into its corresponding donor. Thus an enzyme can be in two possible states, *E_i_* ^(1)^ and *E_i_* ^(0)^, with *E_i_* ^(1)^ + *E_i_* ^(0)^ = *E_i_*. Here, *E_i_* is the total dosage of enzyme *i*, *E_i_* ^(0)^ is the concentration of the enzyme without its group being bound to it, and *E_i_* ^(1)^ is the concentration of enzyme with its group being bound to it. The free energy difference between both states of an enzyme is assumed to be a random value taken from a uniform distribution ranging from zero to one. We further assume that in principle all metabolites that contain a specific biochemical group (i.e., half of the 32 metabolites) can serve as a donor for the transfer reaction involving that group, whereas all metabolites that do not contain that group can in principle serve as an acceptor. We assume linear kinetics for the transfer of a group to an enzyme, given by *v* = *k_ij_*(*E_i_* ^(0)^ *X_(ij)_* ^(1)^−*E_i_* ^(1)^ *X_(ij)_* ^(0)^/*q_ij_*), where *k_ij_* is the rate constant of the reaction *j* of an enzyme *i*, *X_(ij)_* ^(1)^ is the concentration of the donor of the reaction, *X_(ij)_* ^(0)^ is the concentration of the corresponding acceptor, and *q_ij_* is the equilibrium constant of the reaction resulting from the free energies of the reactants. For the transfer of a group from a donor to an acceptor, two half-reactions need to be coupled. This results in Michaelis-Menten–like kinetics and implies that functional enzymes need to maintain nonzero rate constants for at least two reactions that are coupled. Enzymes that maintain nonzero rate constants for more than two reactions are defined as multifunctional. We assume that in the initial network there are 5 enzymes that are unspecific and transform groups from each donor to each acceptor with the same rate constant. The initial dosage of enzymes is *E_i_* = 1.

### Transporters

We assume that transporters transport metabolites passively across the cell membrane. The rate of transport is given by *v* = *T_i_t_ij_*(*X_j_*−*X_jext_*), where *T_i_* is the dosage of the transporter *i*, *t_ij_* is the rate constant for the transport of metabolite *j*, *X_j_* is the metabolite concentration in the cell, and *X_jext_* is the metabolite concentration in the environment. We assume that in the initial network there is a single transporter that transports all metabolites with the same rate constant. The initial dosage of the transporter is *T_0_* = 1.

### Biomass formation, growth, and network fitness

Biomass is formed by the condensation of specific metabolites. The rate of biomass formation follows linear kinetics given by the product *v_BM_* = *k_BM_*Π*_i_X_i_* over all metabolites *X_i_* that are involved in biomass formation. The rate constant is set to *k_BM_* = 1 in all simulations. We assume that the formation of biomass leads to growth. The growth rate is given by *W* = 1/V*dV/*dt* = *v_BM_*/(*C_0_*+*C_E_E*+*C_T_T*), where *C_0_* is the amount of biomass that is required for structural compounds (i.e., those compounds that are not directly involved in cellular metabolism), *C_E_* is the amount of biomass per enzyme, *C_T_* is the amount of biomass per transporter, *E* is the total dosage of enzymes, and *T* is the total dosage of transporters. The parameters *C_0_*, *C_E_*, and *C_T_* are set to 10, 1, and 1, respectively. Note that due to cell growth, metabolites are constantly diluted at a rate equal to the growth rate. The fitness of a network in a given medium is assumed to be proportional to the steady-state growth rate. The fitness in an environment that fluctuates between different media is given by the geometric mean over the fitness values a network has on each of the media.

### Tradeoff between specificity and catalytic activity

We assume that enzymes can either catalyze a large number of reactions with low activity, or a lower number or reactions with improved catalytic activities. Specifically, we assume that the sum Σ*_j_ k_ij_* ^1/*α*^ and Σ*_j_ t_ij_* ^1/*α*^ over all rate constants *k_ij_* or *t_ij_* of an enzyme or transporter, *i*, respectively, is constant. For values of *α*>1, increasing the rate constant for a single reaction has an over-proportional effect on all other rate constants. In our simulations we use Σ*_j_ k_ij_* ^1/*α*^ = 1, Σ*_j_ t_ij_* ^1/*α*^ = 1, and *α* = 2. This implies, for example, that a transporter catalyzing the transport of a single metabolite has a four times higher rate constant for this reaction than a transporter that is specialized on the transport of two metabolites.

The resulting trade-off between enzyme specificity and activity in the model is inline with the general findings from protein engineering and directed evolution experiments [37], [53]. In particular, the tradeoff in our model allows specialized enzymes to retain some (minor) catalytic activity for other reactions. This resembles a situation described as weak negative tradeoff [37]. However, because two specialized enzymes will be better than a single multifunctional enzyme present at double dosage, there is also selection for specialization. The presence of such selection in the model seems justified by the fact that most enzymes in natural metabolic networks are specialized.

In an alternative model, we further relax the assumption of a strong tradeoff between specificity and catalytic activity and allow enzymes to specifically maintain a background activity for all possible reactions. This alternative model allows us to use any network for the starting point of evolutionary simulations. Using this model, we have analyzed whether forcing specialization of enzymes towards specific reactions (which in some extent decreases complexity in the system) has any effect on our conclusions. As shown in Figure S2, we find that this alternative model to produce qualitatively the same results as with the main model.

### Mutations and the course of evolution

We assume that there are two types of mutations: (1) mutations that change the kinetic properties of an enzyme and (2) mutations that change the number of copies of an enzyme, i.e., gene deletions and duplications. For the first type of mutation we assume that the value of *k_ij_* ^1/*α*^ or *t_ij_* ^1/*α*^, respectively, for a single reaction is either increased or decreased by a small value of *m* = 0.05, while the rate constants of the other reactions are decreased, or increased appropriately. Gene deletions and duplications decrease and increase the dosage of an enzyme respectively. To simulate evolution, we first calculate the effect of all possible mutations in the current network on the steady-state growth rate to obtain the mutant with maximal increase in fitness. This mutation is then assumed to become fixed and the resulting network is used to search for the next mutations. Details on the calculation of the steady states are as described in [36]. Gene duplications and deletions are assumed to be rare compared to mutations affecting the catalytic properties of enzymes and transporters and are considered only if none of the mutations affecting kinetic properties are beneficial. We find that relaxing this assumption and considering duplications as frequently as other mutations does not alter the conclusions given in the main text (Figure S1). An evolutionary simulation ends if there are no beneficial kinetic mutations, gene duplications or deletions.

For each set of independent simulations, we randomly chose nutrients, metabolites involved in biomass formation, and the free energies of the metabolites. Changing free energies of the metabolites alters the energetic landscape of the initial network and might favor different pathways even if the topology of the network remains the same.

### Model parameters

Note that, while changing the many parameters of the model could easily alter the properties of individual evolved networks, the qualitative nature of the results presented here would be maintained. This is because our analysis is a comparative one among networks that evolve under different evolutionary scenarios. While model details would change the outcome of evolution in specific simulation, they would do so in similar ways under the different scenarios considered.

Specific parameter choices in this study differ from a previous study using the same model [36], as here we run a large number of simulations of smaller networks. To be able to manage the computational cost of these simulations, we adjusted the parameters for the costs of biomass formation (from 50 to 10), the number of metabolites involved in biomass formation (from 8 to 4), and the mutation size (from 0.01 to 0.05).

### Network robustness

The evolved networks contain many enzymes in multiple copies. These multiple copies of a single enzyme could be seen as isoenzymes. As one would expect for isoenzymes, the knockout of a single copy would have a relatively small effect. We here determine robustness by knocking out all enzymes of the same type. This gives a measure for how essential the reaction catalyzed by an enzyme is.

More specifically, we calculate robustness as network's rate of biomass production (*v_BM_*, see above) in the face of enzyme knockouts. To measure it, we delete each enzyme existing in a given network one by one and calculate the rate of biomass formation for each mutant. This allows us to characterize the full dynamical effect of a gene deletion on biomass formation, rather than just viability (i.e. non-zero vs. zero biomass production rate) and effects on yields.

### Independent metabolic fluxes

To understand the global structure of evolved networks, we measure metabolic flux from metabolites to biomass. In particular, we calculate, for each network, the number of independent fluxes by using the kernel of the stoichiometric matrix derived from that network. The details of this technique is discussed in detail elsewhere [54].

## Supporting Information

Figure S1 Distribution of relative fitness for single knockouts in networks resulting from different evolutionary scenarios and using a model version where duplications are introduced as frequently as small mutations. Each distribution contains measurements from 100 networks and is shown as a boxplot, as implemented in the statistical package “R” (www.r-project.org). See legend of Figure 1 for analysis and naming details.
(2.58 MB TIF)

Figure S2 Distribution of relative fitness for single knockouts in networks resulting from different evolutionary scenarios and using a model version where enzymes are forced to maintain a non-zero rate for all possible reactions. The simulation scheme is also changed from the original analysis; for the fluctuating environment scenario, we have used networks evolved under stable environments as the starting network. This corresponds to modelling a shift in the environment from stable source to fluctuating sources of metabolites. Each distribution contains measurements from 20 networks and is shown as a boxplot, as implemented in the statistical package “R” (www.r-project.org). See legend of Figure 1 for analysis and naming details.
(2.55 MB TIF)
