# Supplementary figures and images for: Evolution under Fluctuating Environments Explains Observed Robustness in Metabolic Networks
Source: PLoS Comput Biol. 2010 Aug 26;6(8):e1000907. doi: 10.1371/journal.pcbi.1000907 (PMC2928748; doi:10.1371/journal.pcbi.1000907)

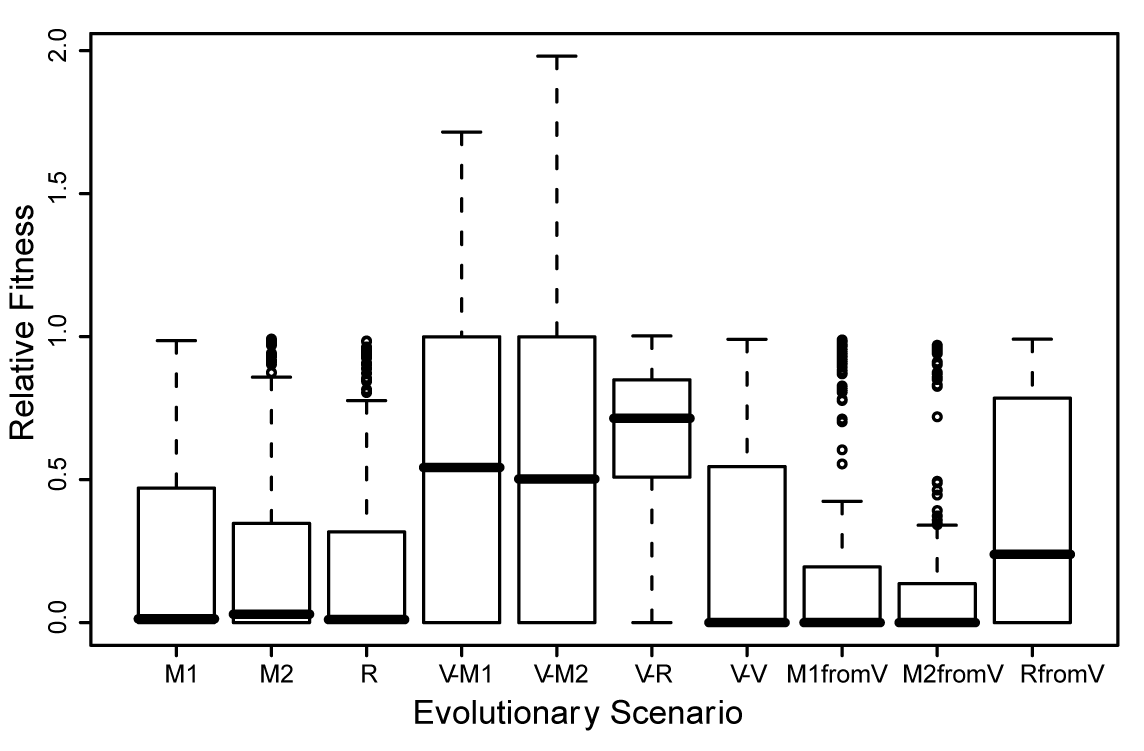

Supplement: Figure S1 — Distribution of relative fitness for single knockouts in networks resulting from different evolutionary scenarios and using a model version where duplications are introduced as frequently as small mutations. Each distribution contains measurements from 100 networks and is shown as a boxplot, as implemented in the statistical package “R” (www.r-project.org). See legend of Figure 1 for analysis and naming details. (2.58 MB TIF) [file pcbi.1000907.s001.tif]

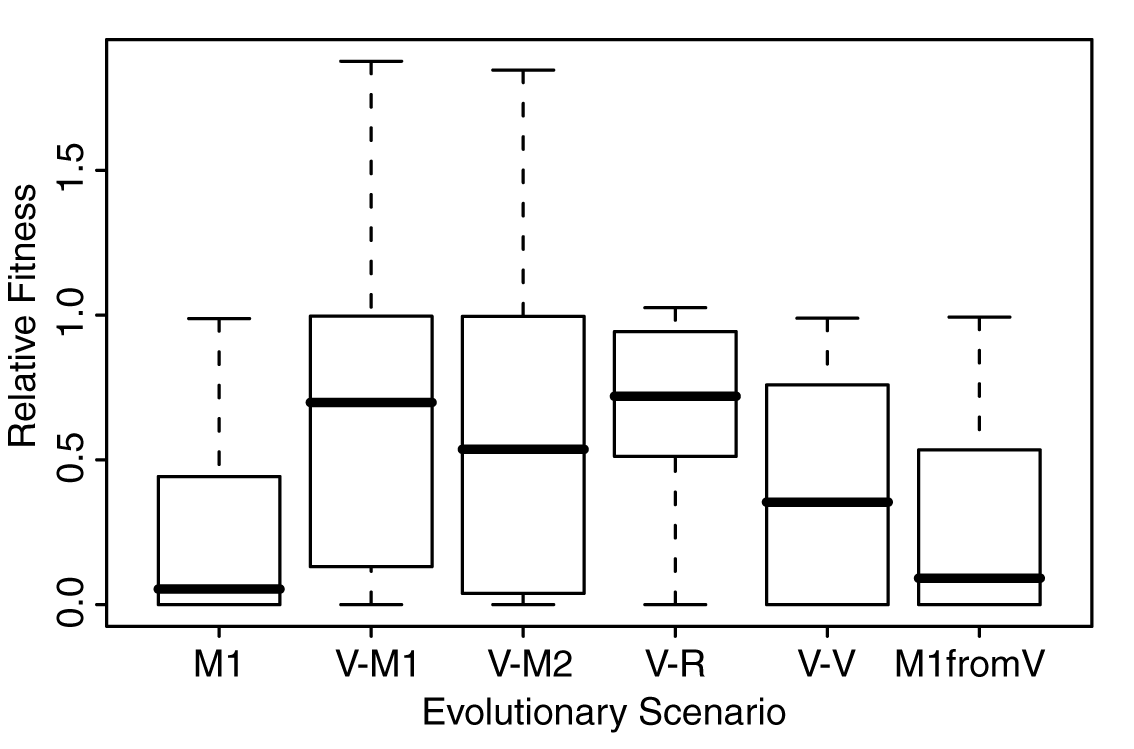

Supplement: Figure S2 — Distribution of relative fitness for single knockouts in networks resulting from different evolutionary scenarios and using a model version where enzymes are forced to maintain a non-zero rate for all possible reactions. The simulation scheme is also changed from the original analysis; for the fluctuating environment scenario, we have used networks evolved under stable environments as the starting network. This corresponds to modelling a shift in the environment from stable source to fluctuating sources of metabolites. Each distribution contains measurements from 20 networks and is shown as a boxplot, as implemented in the statistical package “R” (www.r-project.org). See legend of Figure 1 for analysis and naming details. (2.55 MB TIF) [file pcbi.1000907.s002.tif]
